# Bibliometric analysis of global research trends in spatially fractionated radiotherapy

**DOI:** 10.3389/fonc.2026.1828039

**Published:** 2026-05-08

**Authors:** Lingling Meng, Yupeng Di, Lin Ma, Baolin Qu

**Affiliations:** 1 Department of Radiation Oncology, Senior Department of Oncology, The First Medical Center of PLA General Hospital, Beijing, China; 2 Medical School of Chinese PLA, Beijing, China; 3 Department of radiation protection medicine, School of Preventive Medicine, Fourth Military Medical University, Xi’an, China; 4 Department of Radiation Oncology, Air Force Medical Center, PLA, Beijing, China

**Keywords:** bibliometric analysis, clinical translation, research trends, SFRT, spatially fractionated radiotherapy, thematic evolution

## Abstract

**Background:**

Spatially Fractionated Radiotherapy (SFRT) is an innovative radiation oncology technique that strategically redistributes doses within a tumor volume to optimize tumor ablation while preserving normal tissue integrity. Despite substantial technological advancements and growing interdisciplinary interest, a systematic characterization of the global research environment remains absent. This study aims to provide a comprehensive bibliometric evaluation to map research trajectories and identify future priorities.

**Methods:**

A systematic search was performed across the Web of Science Core Collection (WoSCC) and PubMed databases, covering the period from January 1, 2005, to December 31, 2025. Following the standard PRISMA guidelines, 427 eligible publications (385 primary articles and 42 reviews) were selected from an initial pool of 1,221 records. Three analytical platforms—VOSviewer, CiteSpace, and Biblioshiny—were integrated to synthesize data on publication trends, institutional contributions, collaborative networks, and thematic evolution.

**Results:**

The SFRT landscape demonstrates a distinct three-phase evolutionary pattern, characterized by a substantial acceleration in publications and citations from 2020 to 2025. The United States maintains the highest cumulative research output, whereas France exhibits leading institutional productivity and significant citation impact. Foundational works in physical dosimetry and early preclinical models have established the disciplinary knowledge base. Thematic evolution identifies five core clusters, revealing a paradigm shift from early investigations into microbeam technology and cellular radiobiology toward recent advancements in proton minibeam therapy, computational precision, and clinical feasibility assessment.

**Conclusion:**

SFRT is a maturing interdisciplinary field that increasingly integrates dose heterogeneity with complex radiobiological responses. To facilitate broader clinical implementation, future research must focus on identifying robust biological markers, refining delivery technologies, and establishing standardized protocols through large-scale prospective trials.

## 1 Introduction

Spatially Fractionated Radiotherapy (SFRT) is an innovative radiation oncology technique that strategically redistributes radiation dose within a tumor volume, creating areas of high-dose ‘peaks’ interspersed with lower-dose ‘valleys’ (1–3). This approach aims to maximize tumor ablation through direct cytotoxic effects in high-dose regions and indirect radiobiological mechanisms (such as bystander and abscopal effects, and vascular disruption) in low-dose areas, while simultaneously enhancing normal tissue sparing by exposing only a partial volume to high doses (3, 4). Emerging as a promising paradigm for treating radioresistant or bulky tumors, SFRT represents a significant evolution from conventional homogeneous dose delivery, offering a potentially superior therapeutic index (5).

The rapid advancement of SFRT reflects the convergence of medical physics, radiobiology, immunology, and oncology, driving exponential growth in a body of literature characterized by diverse methodologies and varying levels of clinical application (4). Systematic analysis is essential to identify trends, assess research impact, and guide future priorities. The absence of detailed bibliometric analyses on SFRT leads to knowledge gaps that hinder effective research planning (6).

Bibliometric analysis provides powerful tools for systematically mapping scientific landscapes, identifying trends, and predicting future directions through co-citation analysis, keyword co-occurrence mapping, and temporal trend analysis. Such approaches are particularly valuable in rapidly advancing interdisciplinary fields where traditional reviews struggle to capture full complexity (7).

Therefore, we conducted a systematic bibliometric investigation of the global research landscape spanning two decades (2005–2025). This study quantitatively analyzes publication trends, identifies productive countries and institutions, maps collaboration networks, examines thematic evolution, and projects future directions. Through rigorous application of multiple analytical frameworks, we provide the first comprehensive map of this rapidly expanding field, offering essential guidance for optimizing research investments and clinical implementation strategies.

## 2 Materials and methods

### 2.1 Selection of databases and search methodology

### 2.1.1 Database rationale and identification

This investigation utilized the Web of Science Core Collection (WoSCC) and PubMed databases to identify scholarly publications from January 1, 2005, to December 31, 2025.

The search strategy employed specialized SFRT terminology optimized for the technical requirements of each platform (**Supplementary Figure 1**).

#### 2.1.2 Synergistic data retrieval

A dual-database approach was implemented to ensure maximum dataset fidelity (8, 9). WoSCC offers distinct benefits, including standardized citation metrics and high metadata compatibility with advanced analytical tools such as VOSviewer and CiteSpace. Concurrently, the inclusion of PubMed ensures the exhaustive retrieval of pivotal biomedical literature, capturing clinical trials and specific medical insights that might be under-represented in broader indices.

The strategic consolidation of these repositories maximizes analytical precision and research depth by mitigating platform-specific indexing biases.

### 2.2 Selection parameters

Inclusion criteria: (i) Academic publications comprising original research articles and comprehensive review papers published between 2005 and 2025; (ii) Research specifically investigating SFRT. Exclusion criteria include: (i) non-substantive document types such as editorial materials, meeting abstracts, and letters; (ii) studies with minimal or no direct relevance to SFRT.

### 2.3 Process of literature evaluation and refinement

The data identification and selection process were conducted in accordance with the PRISMA guidelines to ensure methodological rigor. As illustrated in the standard flow diagram (**Supplementary Figure 1**), the initial database search generated a total of 1,221 records (614 from WoSCC and 607 from PubMed). After the removal of 594 duplicate records, 627 unique publications underwent preliminary screening.

Two independent researchers (Yupeng Di and Lingling Meng) manually screened the titles, abstracts, and, when necessary, the full texts of the 627 remaining records to ensure absolute relevance to the SFRT research domain. Any inconsistencies during the evaluation were resolved through consultation and consensus with the corresponding author.

Following this rigorous refinement, 200 records were excluded based on predefined criteria. Specifically, 194 meeting abstracts, 3 editorial materials, 2 letters, and 1 non-English record were identified as unsuitable for systematic bibliometric analysis.

The final curated dataset comprised 427 high-quality scholarly publications, including 385 original research articles and 42 comprehensive reviews. Detailed statistics for each stage of identification, exclusion, and final inclusion are documented in **Supplementary Figure 1** to enhance thematic transparency and reproducibility.

### 2.4 Frameworks for bibliometric analysis

#### 2.4.1 Methodological integration and rationale

To ensure a rigorous and multi-dimensional characterization of the Spatially Fractionated Radiotherapy (SFRT) landscape, this study integrated three advanced bibliometric platforms: VOSviewer, CiteSpace, and Biblioshiny. The synergystic use of these tools is necessitated by their complementary analytical strengths, which individually would be insufficient to capture the full structural and temporal complexity of the field.

VOSviewer is optimized for constructing and visualizing intricate bibliographic networks based on distance metrics. CiteSpace facilitates the identification of disciplinary knowledge transfer through dual-map overlays and detects emergent research fronts via citation burst algorithms. Biblioshiny, an R-based package, provides robust quantitative mapping of thematic evolution and longitudinal tracking of research priorities. The combination of these frameworks enhances the statistical reliability and transparency of the findings (6, 10).

#### 2.4.2 VOSviewer (version 1.6.17)

VOSviewer was primarily employed to create structural visualizations of the intellectual environment. This included the construction of collaborative maps across geographical, institutional, and individual scholarly domains.

The software also facilitated in-depth co-citation analyses of academic publications and a keyword co-occurrence network. In these visualizations, node scaling indicates publication volume, while color differentiation represents cluster patterns or chronological shifts. The pathways between nodes illustrate the intensity of collaborative or citation-based interconnections (11).

#### 2.4.3 CiteSpace (version 6.1.R6)

CiteSpace was utilized to perform dual-map overlay analyses, which visualize the citation trajectories across disparate academic disciplines, revealing how clinical medicine integrates principles from physics and molecular biology.

Additionally, CiteSpace was used to execute citation burst detection. This algorithm identifies publications or keywords that experience a significant surge in frequency within a specific timeframe, serving as a critical indicator of emergent research trends and shifting paradigms in SFRT (7, 12, 13).

#### 2.4.4 Biblioshiny (R-package bibliometrix, version 5.2.0)

Biblioshiny served as the core engine for quantitative and longitudinal analysis. It was utilized to map the global distribution of scholarly production and to evaluate the dynamics of international collaboration networks.

Crucially, this tool enabled the construction of thematic evolution maps, allowing for the systematic tracking of how research focus has transitioned from high-dose physical delivery to complex biological and immunological modulation over the specified two-decade period (10, 14, 15).

#### 2.4.5 Principles of co-citation analysis

Co-citation analysis identifies intellectual relationships by examining the frequency with which two documents are cited together in subsequent literature. This proximity indicates thematic relatedness and intellectual overlap within the research domain.

In this study, co-citation analysis was applied to identify the core knowledge base and foundational works that underpin current SFRT research fronts. By quantifying the strength of these relationships, we visualized the intellectual structure and the evolutionary pathways that have shaped the current therapeutic strategies in the field.

### 2.5 Data processing and visualization

Microsoft Office Excel 2019 was used for quantitative data analysis, including calculation of publication trends, citation metrics, and collaborative indices. Polynomial fitting was applied to publication data to project future research trends.

To ensure transparency and reproducibility, rigorous standardization protocols were applied before network generation. Synonymous keywords (e.g., “SFRT,” “spatially fractionated radiotherapy,” and “grid therapy”) were merged into standardized terms. The detailed keyword consolidation dictionary is provided in **Supplementary Table 1**.

Furthermore, the key configuration parameters used for the respective analytical software—such as time slicing intervals and network pruning algorithms (e.g., Pathfinder) in CiteSpace, as well as minimum occurrence thresholds in VOSviewer—are comprehensively detailed in **Supplementary Table 2**. All visualizations were standardized for clarity across different tools.

## 3 Results

### 3.1 Analysis of publication quantities

**Figure 1:**
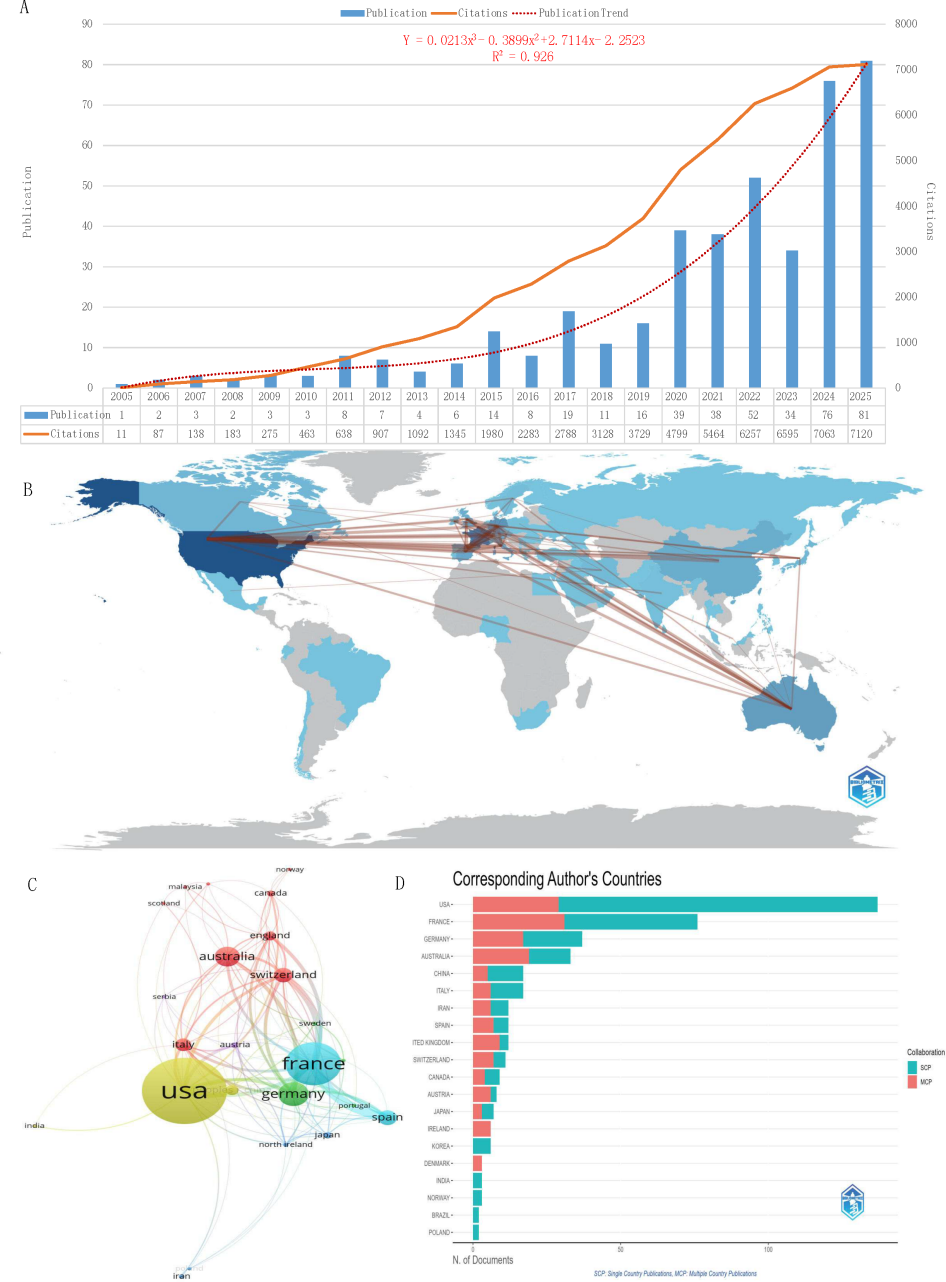
Annual publication and citation trends, country contributions, and collaboration networks in SFRT research. **(A)** Annual research output on SFRT. The orange curve represents publication trends, while the green curve indicates citation trends. The red dashed trendline is fitted to the number of publications using a cubic polynomial model generated with Microsoft Office Excel 2019. **(B)** Geographic distribution map of contributions created using the Biblioshiny online platform. **(C)** Inter-country collaboration network diagram generated using VOSviewer software. This study included 25 countries based on the criterion “≥4 publications per country” (attraction/repulsion values: 1/-3). **(D)** Geospatial distribution map illustrating the countries of corresponding authors visualized using the Biblioshiny online platform.

This study analyzed 427 SFRT-related publications, encompassing 51 countries, 497 research institutions, and 1,819 authors across 92 journals. **Figure 1A** shows the annual publication and citation trends from 2005 to 2025. The research field exhibits a distinct three-phase evolution. The initial phase (2005–2014) saw minimal research activity. Between 2015 and 2019, there was a phase of stable development marked by consistent publication growth and an increase in citation rates.

3.1.2 The current phase (2020–2025) has seen a substantial acceleration in publication output and citation frequency. Publication and citation counts approximately doubled from 2020 to their peak. This indicates increased research output coupled with a significant rise in research impact and academic recognition. The polynomial trendline (R² = 0.926) suggests that this robust expansion signifies a fundamental shift towards mainstream academic recognition of the field. The polynomial fitting results suggest continued high activity in this field, with projected annual publication volumes of 96 and 113 papers for 2026 and 2027, respectively. The average annual growth rate from 2018 to 2025 is approximately 24.57%. This further confirms the field’s trajectory of continuous steady development. After elucidating the core growth patterns and future trends in this field, we will explore the geographic distribution and institutional contributions propelling research expansion.

### 3.2 Analysis of countries and institutions

In the analysis of international collaboration, **Figures 1B–D** illustrate the primary contributing countries and their collaborative networks in the SFRT field. The United States tops the list with 137 publications, more than double France’s 76 publications (**Table 1**). The United States has a moderate average citation rate of 21.01 per publication, reflecting a significant amount of research with limited impact. Switzerland exhibits a significant research impact, averaging 18.09 citations per publication across just 11 publications, indicating its work is highly influential and frequently cited despite a smaller output. The geographic diversity in research is exemplified by established Western hubs such as the United States, France, Germany, Australia, Italy, Spain, the UK, and Switzerland, major Asian countries including China, Iran, Japan, and India, as well as emerging research economies like Malaysia and Serbia. This diversity highlights the worldwide importance and clinical necessity of SFRT research.

**Table 1:** The top 10 countries related to SFRT.

Rank	Country	Counts	Citation	Average citation/publications
1	The United States	137	2879	21.01
2	France	76	1788	23.53
3	Germany	37	417	11.27
4	Australia	33	590	17.88
5	China	17	110	6.47
6	Italy	17	233	13.71
7	Iran	12	31	2.58
8	Spain	12	120	10.00
9	The United Kingdom	12	127	10.58
10	Switzerland	11	199	18.09

**Figure 2:**
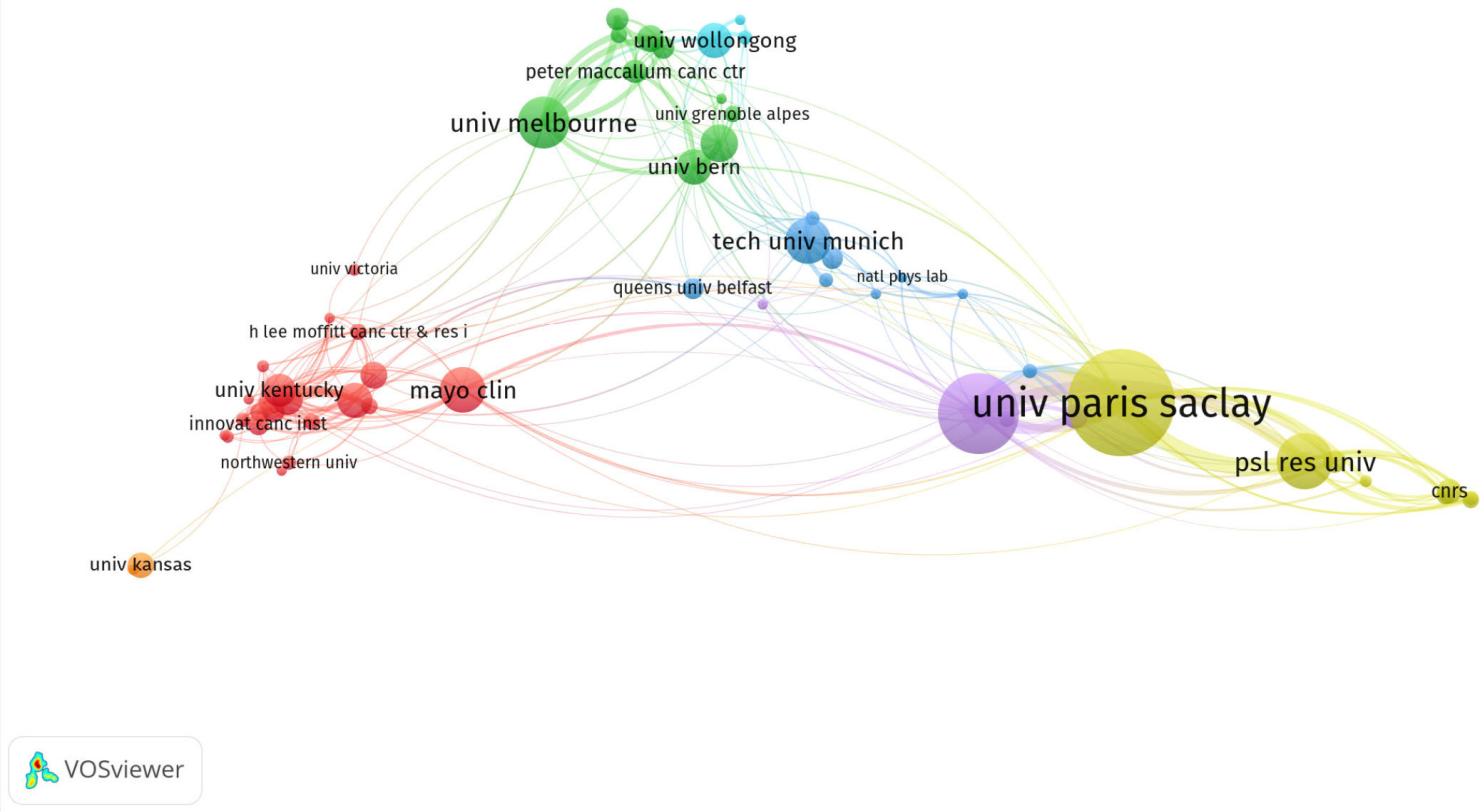
Visualization of institutions in SFRT research constructed using VOSviewer software. This study selected 58 institutions based on a minimum of 5 publications for visualization, and constructed a collaborative network based on the number and relationship of publications of each institution (attraction/repulsion values: 1/-2).

Institutional analysis reveals that France occupies a dominant position in this field, with four of the ten most productive institutions originating from the country (**Figure 2**; **Table 2**). The University of Paris-Saclay tops the rankings with 52 papers and 802 citations, ahead of the University of Paris with 39 papers and 478 citations, and Shanghai Jiao Tong University with 21 papers and 387 citations. However, research impact metrics reveal significant disparities in citations and publications: The University of Arkansas for Medical Sciences, for example, achieved a remarkably high citation frequency (558 citations) despite having the lowest publication count (17 papers). Similarly, the University of Wollongong published 17 papers yet achieved a citation frequency of 107. Citation performance among U.S. institutions showed significant variation, with the Mayo Clinic receiving 254 citations and the University of Arkansas for Medical Sciences receiving 558 citations.

**Table 2:** The top 10 organization related to SFRT.

Rank	Organization	Counts	Citations
1	University of Paris-Saclay (France)	52	802
2	Université PSL (France)	39	478
3	PSL Research University (France)	27	762
4	University of Melbourne (Australia)	25	463
5	Technical University of Munich (Germany)	22	348
6	Mayo Clinic (USA)	22	254
7	European Synchrotron Radiation Facility (France)	18	550
8	University of Arkansas for Medical Sciences (USA)	17	558
9	University of Bern (Switzerland)	17	363
10	University of Wollongong (Australia)	17	107

### 3.3 Leading and co-cited journals

#### 3.3.1 Journal publication and citation metrics

**Figure 3:**
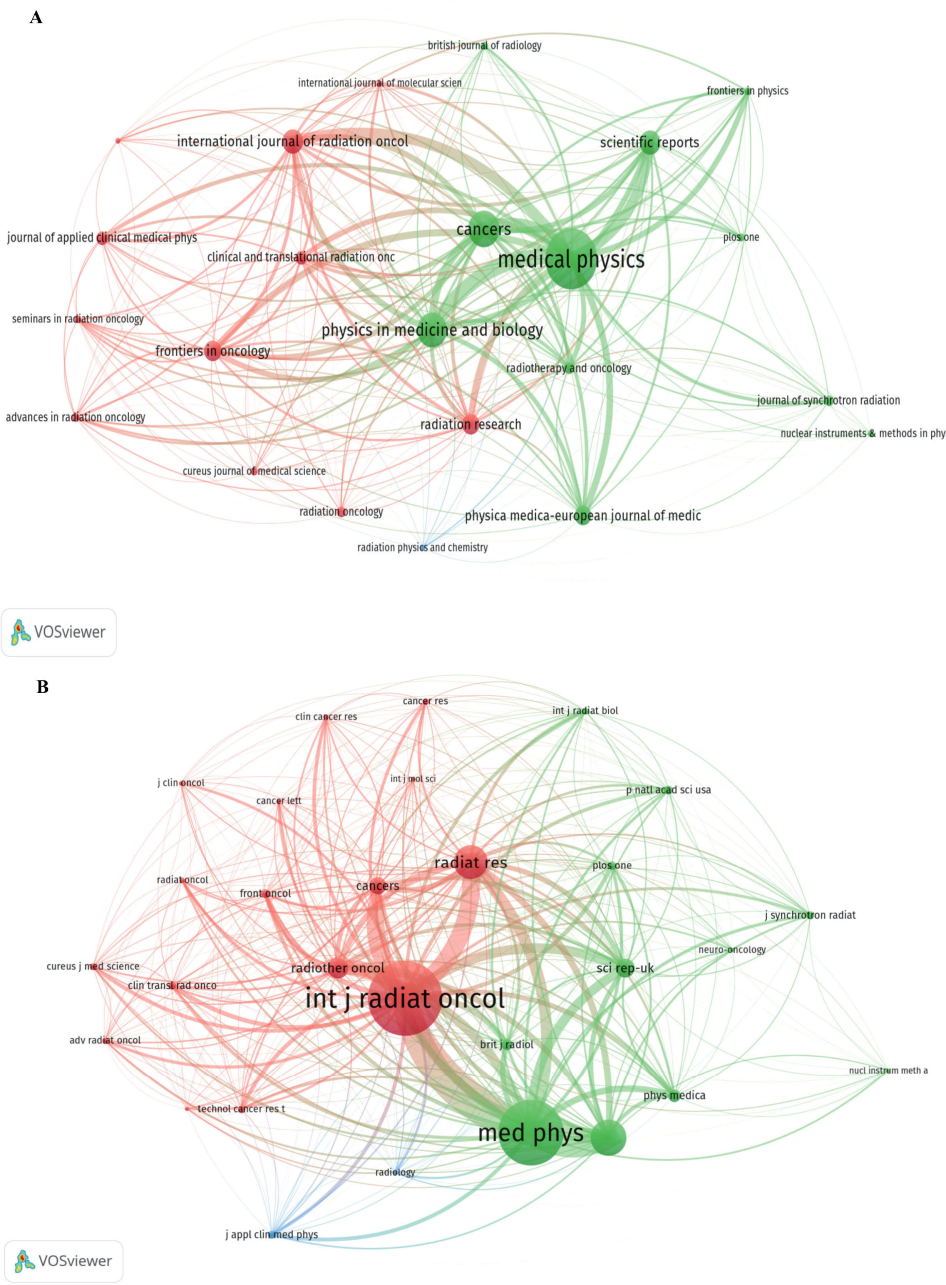
Visualization of journals **(A)** and co-cited journals **(B)** in SFRT research generated using VOSviewer software. **(A)** This study enrolled 23 journals based on a minimum of 5 relevant publications and mapped the journal network (attraction/repulsion values: 2/-2). **(B)** More than 100 co-citation journals (29 journals) were filtered to map the co-citation network (attraction/repulsion values: 2/-2).

The 427 SFRT-related publications analyzed in this study were distributed across 92 distinct journals. Medical Physics led in publication volume with 53 papers and 1,090 citations [Impact Factor (IF): 3.2], followed by Cancers with 32 papers and 462 citations (IF: 4.4), and Physics in Medicine and Biology with 30 papers and 232 citations (IF: 3.4) (**Figure 3A**; **Table 3**). The International Journal of Radiation Oncology • Biology • Physics demonstrated the highest citation-to-publication ratio of 42.1 (21 papers, 884 citations). Conversely, Physics in Medicine and Biology recorded a lower average citation rate of 7.7 citations per paper despite its high publication volume.

**Table 3:** The top 10 journals related to SFRT.

Rank	Journal	Counts	Citations	IF^a^	Q^b^
1	Medical Physics	53	1090	3.2	1
2	Cancers	32	462	4.4	2
3	Physics in Medicine and Biology	30	232	3.4	1
4	International Journal of Radiation Oncology • Biology • Physics	21	884	6.5	1
5	Scientific Reports	21	478	3.9	1
6	Frontiers in Oncology	18	147	3.3	2
7	Radiation Research	18	639	2.7	2
8	Physica Medica	17	228	2.7	2
9	Clinical and Translational Radiation Oncology	12	294	2.7	2
10	Radiotherapy and Oncology	10	144	5.3	1

^a^The impact factor of the journal are obtained from Journal Citation Reports 2025.

^b^The quartile of the journal are obtained from Journal Citation Reports 2025.

#### 3.3.2 Co-citation analysis of journals

Co-citation analysis mapped the foundational knowledge sources cited by the SFRT dataset. The International Journal of Radiation Oncology • Biology • Physics (2,079 co-citations) and Medical Physics (1,827 co-citations) emerged as the most frequently co-cited journals. The network also prominently featured general multi-disciplinary journals, including Physics in Medicine and Biology (995 co-citations) and Radiation Research (948 co-citations) (**Figure 3B**; **Table 4**). Additionally, oncology-focused journals such as Radiotherapy and Oncology (574 co-citations, IF: 5.3) and Cancers (478 co-citations, IF: 4.4) maintained significant co-citation frequencies within the network.

**Table 4:** The top 10 co-cited journals related to SFRT.

Rank	Journal	Co-citations	IF^a^	Q ^b^
1	International Journal of Radiation Oncology • Biology • Physics	2079	6.5	1
2	Medical Physics	1827	3.2	1
3	Physics in Medicine and Biology	995	3.4	1
4	Radiation Research	948	2.7	2
5	Radiotherapy and Oncology	574	5.3	1
6	Scientific Reports	522	3.9	1
7	Cancers	478	4.4	2
8	Physica Medica	341	2.7	2
9	British Journal of Radiology	308	3.4	1
10	Frontiers in Oncology	246	3.3	2

^a^The impact factor of the journal are obtained from Journal Citation Reports 2025.

^b^The quartile of the journal are obtained from Journal Citation Reports 2025.

#### 3.3.3 Dual-map overlay and citation pathways

**Figure 4:**
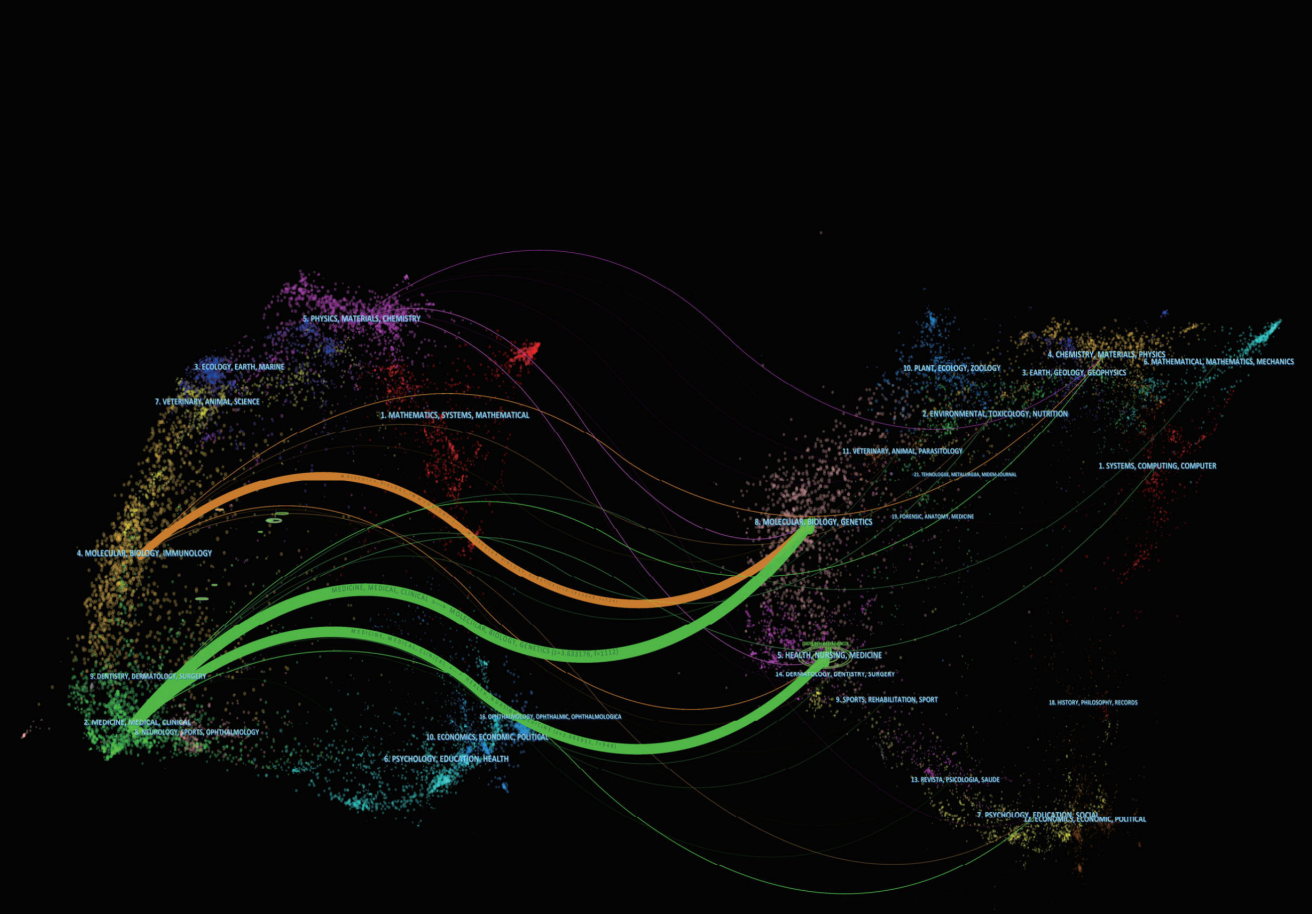
Dual-map overlay of journals related to SFRT created using CiteSpace software.

**Figure 4** presents a dual-map overlay illustrating the macroscopic citation pathways across diverse academic disciplines within SFRT research. The purple curve traces the citation of cited core literature in Chemistry, Materials, Physics, and Molecular Biology/Genetics by citing articles mapped to Physics, Materials, and Chemistry fields. The orange curve represents citation pathways originating from Molecular/Biology/Immunology research directed toward resources in Chemistry/Materials/Physics and Molecular/Biology/Genetics. Furthermore, the prominent green curve visualizes the robust knowledge transfer from cited references in Molecular/Biology/Genetics to citing research within the Medicine, Medical, and Clinical domains.

#### 3.4 Leading and co-cited authors

**Figure 5:**
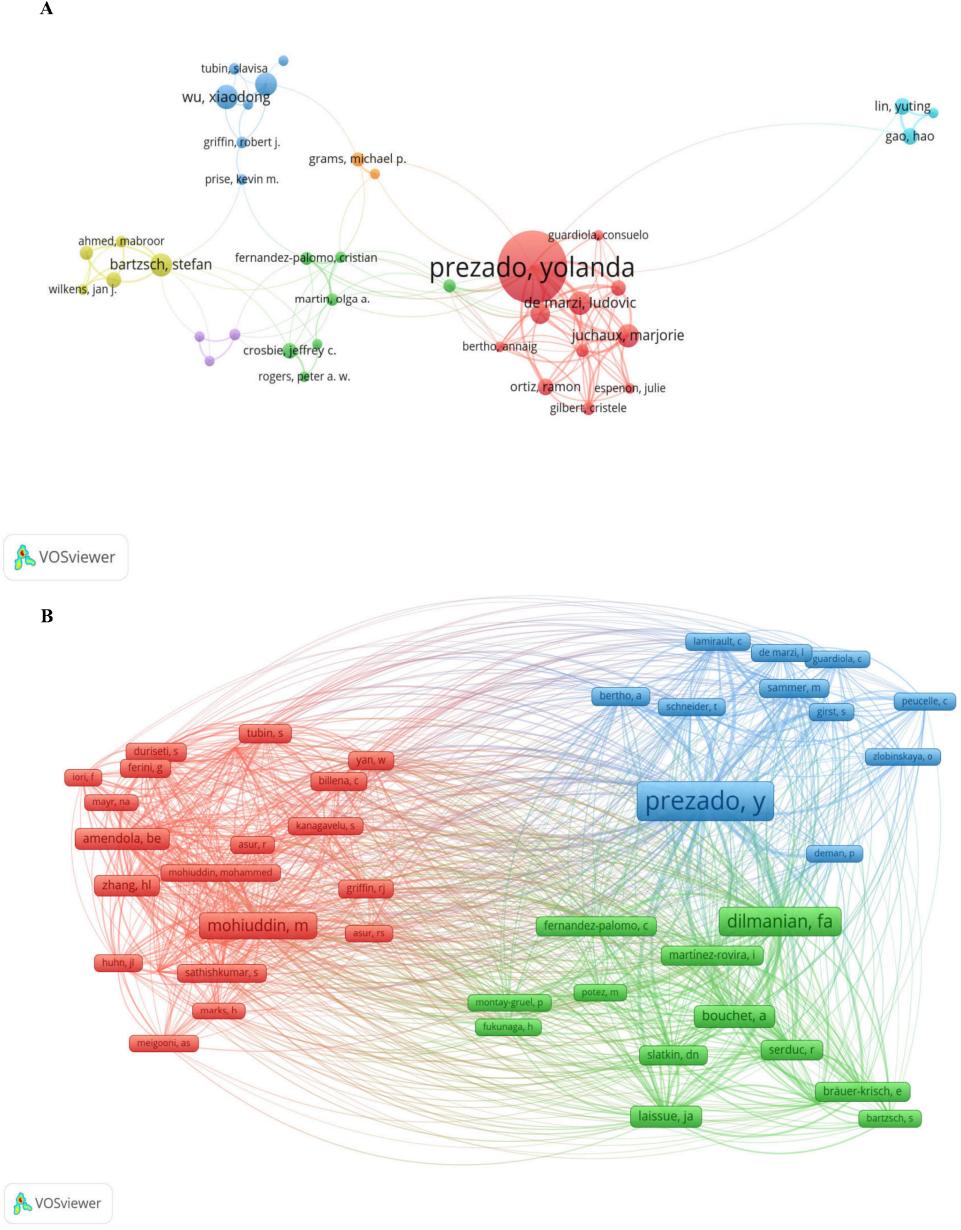
Visualization of authors **(A)** and co-cited authors **(B)** in SFRT research constructed using VOSviewer software. **(A)** A collaborative network was constructed based on 38 researchers whose number of published documents is greater than or equal to 8 (attraction/repulsion values: 1/-6). **(B)** This study selected 48 authors to map the co-citation network based on a minimum of 50 co-citations (attraction/repulsion values: 2/0).

The author landscape in the SFRT research field is characterized by a geographically diverse yet hierarchically stratified network of contributors. Yolanda Prezado has achieved a significant leadership role, having published 78 papers with 1,822 citations and maintaining an ideal H-index correlation (16, 17) (**Figure 5A**; **Table 5**). A distinct pattern emerges within the secondary author group. Six authors stand out: Ludovico De Marzi, Xiaodong Wu, Marjorie Zhu Xiao, Stefan Batzsch, Hualin Zhang, and Annalisa Patriarca. Each has published over 15 papers, yet citation counts vary significantly: Batzsch received 221 citations, while Wu received 803. All possess H-indexes exceeding 10, establishing them as core contributors to SFRT research. Hao Gao and Yuting Lin have identical publication and citation counts (13 papers cited 80 times), which suggests potential collaboration or affiliation with the same institution (**Figure 5A**).

**Table 5:** The top 10 authors related to SFRT.

Rank	Author	Counts	Citations	H-index
1	Prezado, Yolanda	78	1822	25
2	De Marzi, Ludovic	19	308	11
3	Wu, Xiaodong	19	803	12
4	Juchaux, Marjorie	18	523	10
5	Bartzsch, Stefan	18	221	10
6	Zhang, Hualin	18	401	12
7	Patriarca, Annalisa	16	556	12
8	Ortiz, Ramon	13	198	6
9	Gao, Hao	13	80	4
10	Lin, Yuting	13	80	4

The co-citation author network map successfully visualizes the core knowledge structure of the SFRT field. It reveals the status of three authors, Prezado Y., Dilmanian F. A., and Mohiuddin M., as the three core intellectual contributors, with three major academic clusters formed around them. These clusters represent distinct knowledge sources and collaborative networks within SFRT research, collectively making significant contributions to the foundational knowledge base of the field, while also demonstrating internal academic exchange and integration through mutual citations. The node sizes distinctly represent the hierarchical influence levels of various authors within the SFRT field (**Figure 5B**; **Supplementary Table 3**). The distribution of co-citation frequencies, ranging from 667 for Prezado Y. to 126 for Fernandez-Palomo C., indicates significant differences in influence among these foundational contributors.

Analyzing the most frequently co-cited references uncovers the foundational works that have significantly influenced the intellectual evolution of SFRT.

### 3.5 Leading co-cited references

#### 3.5.1 Top co-cited publications and link strengths

**Figure 6:**
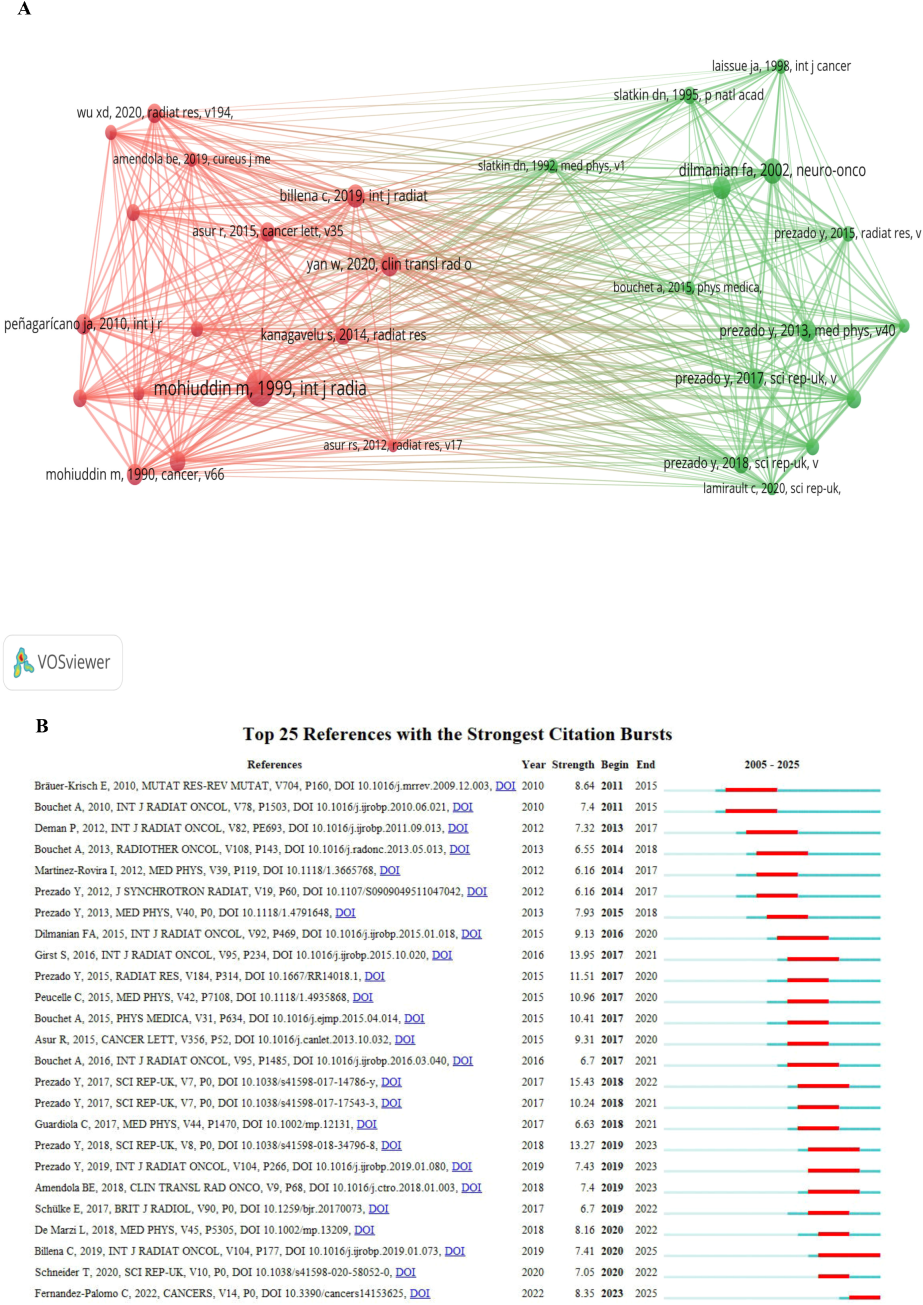
Visualization of SFRT research. **(A)** Co-cited references generated using VOSviewer software. The collaborative network was constructed based on 30 references whose number of co-citations is greater than or equal to 50 (attraction/repulsion values: 1/-1). **(B)** Top 25 references with strong citation bursts created using CiteSpace software. A red bar indicates high citations in that year [this study used the g-index (k = 25) for node selection and applied a time slicing range from 2005 to 2025 (1-year per slice) to analyze research trends].

In the co-cited reference analysis, Mohiuddin et al. (1999, International Journal of Radiation Oncology • Biology • Physics, v45, p721) recorded the highest co-citation count of 144. This publication also exhibited the most significant citation burst, achieving a total link strength of 911 (**Figure 6A**; **Supplementary Table 4**).

#### 3.5.2 Secondary highly cited references and temporal bursts

The second most frequently co-cited reference was Dilmanian et al. (2002, *Neuro-Oncology*, v4, p26) with 94 citations and a total link strength of 551. This was followed by Billena et al. (2019, International Journal of Radiation Oncology • Biology • Physics, v104, p177), which accumulated 88 citations. The distribution of citation bursts, detailing the duration and temporal intensity of these foundational publications, is further illustrated in the top 25 references with strong citation bursts (**Figure 6B**).

### 3.6 Clustering and integration of research domains

The identified keyword clusters illustrate a thorough and interconnected approach to SFRT research (**Supplementary Table 5**). Cluster 1 establishes the foundational technological and radiobiological frameworks of Microbeam Radiation Therapy (MRT), encompassing concepts like dosimetry, specific radiation sources (e.g., synchrotron, x-rays), and their effects on preclinical tumor models such as 9L gliosarcoma in the brain (18, 19). Cluster 2 focuses on the clinical translation and biological modulation of various SFRT techniques, including GRID therapy and lattice radiotherapy (20, 21). It highlights their application in different cancers (e.g., lung cancer), their potential interaction with the immune system (immunotherapy, bystander effect), and their advancement towards clinical implementation, as evidenced by Phase I trials (22–24). Cluster 3 represents the advanced development of proton-based spatially fractionated radiation therapy, particularly Proton Minibeam Radiation Therapy (pMBRT) (25–27). It integrates sophisticated computational modeling techniques, such as Monte Carlo simulations and tools like TOPAS, to optimize beam delivery and understand effects in models like glioma-bearing rats (28, 29). Cluster 4 encompasses the methodological aspects of SFRT, focusing on the feasibility and optimization of various radiation approaches, including proton therapy and general spatial fractionation techniques (5, 30, 31). Cluster 5 addresses the fundamental radiobiological interactions, represented by ‘cells’ and ‘ionizing-radiation’, which underpin all spatially fractionated radiation modalities (18, 32).

These clusters collectively represent a progression from the fundamental radiobiology of ionizing radiation and advanced physical delivery techniques, through their preclinical validation and sophisticated computational modeling, towards clinical translation and the exploration of biological and immunological responses in SFRT (4, 16, 33). These five clusters encapsulate the present research landscape of SFRT. We analyzed temporal trends and thematic development patterns to comprehend the evolution of this landscape.

### 3.7 Evolution over time and thematic development pathway

**Figure 7:**
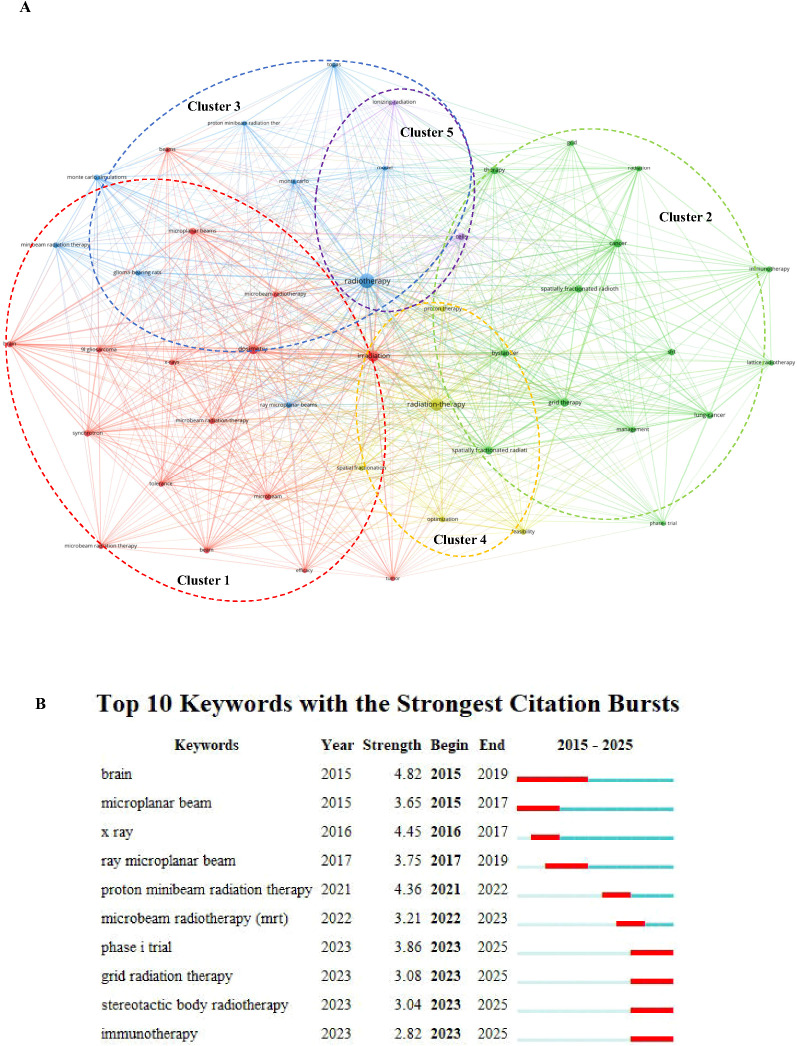
Keyword visualization analysis. **(A)** Keyword cluster analysis using VOSviewer software [more than 15 occurrences (50 keywords) were filtered to map the network] (attraction/repulsion values: 1/-1). **(B)** Top 10 keywords with strongest citation bursts generated using CiteSpace software [this study used the g-index (k = 5) for node selection and applied a time slicing range from 2015 to 2025 (1-year per slice) to analyze research trends].

**Figure 8:**
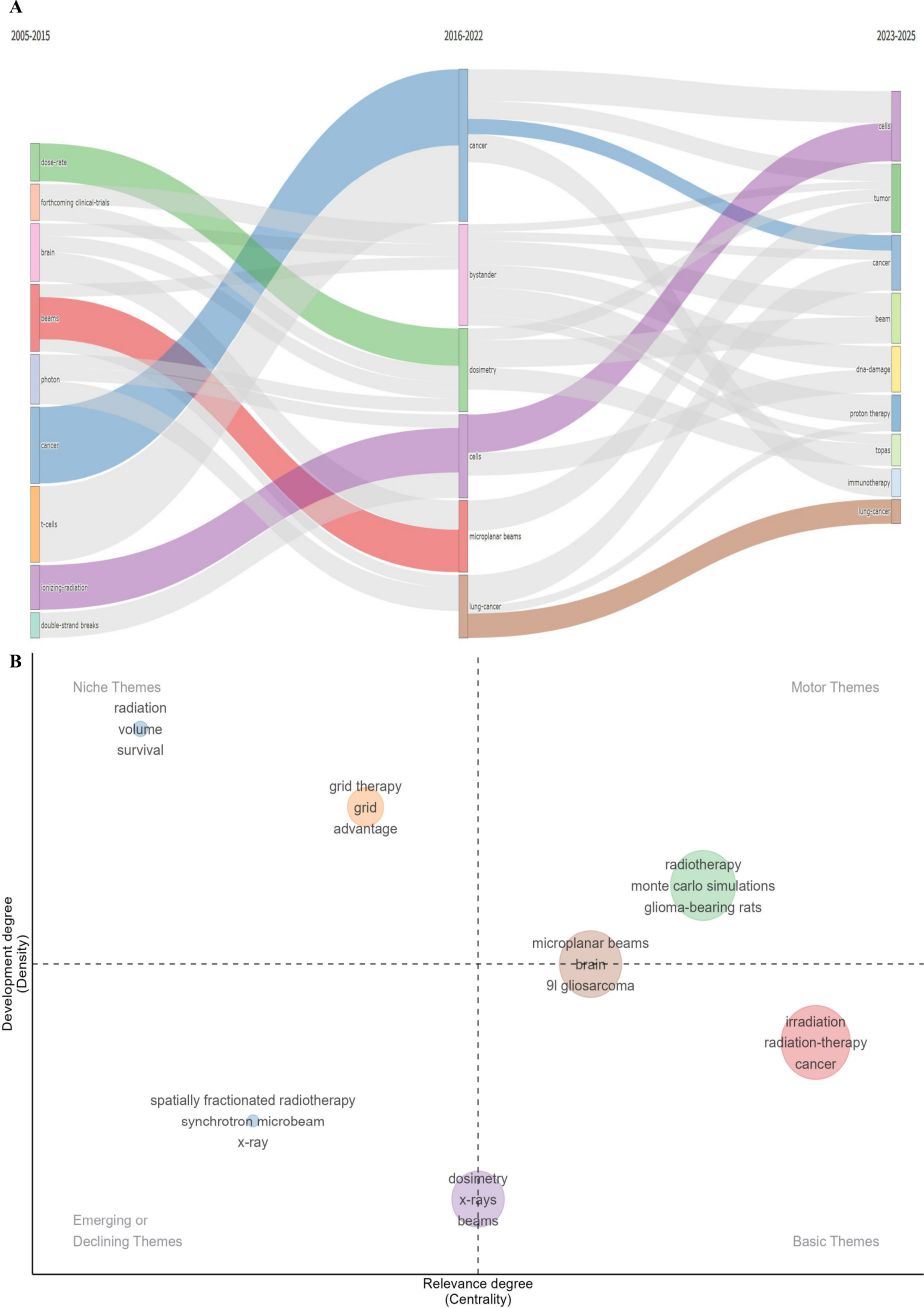
Visualization of SFRT research. **(A)** Thematic evolution on SFRT created using Biblioshiny online platform. **(B)** Thematic map constructed using Biblioshiny online platform: The horizontal axis represents mediating centrality, indicating the theme’s relevance to the field, while the vertical axis represents density, showing how well the theme has developed. Quadrant 1 (top right) represents MOTOR themes, which are both important and well-developed. Quadrant 2 (top left) represents HIGHLY DEVELOPED and INDIVIDUAL themes—those that are well-developed but less relevant to the current domain. Quadrant 3 (bottom left) includes EMERGING OR DECLINING themes, which are underdeveloped and may be either gaining or losing relevance. Quadrant 4 (bottom right) represents BASIC and TRANSVERSAL themes, which are important to the field but not yet well-developed.

The bibliometric analysis from 2005 to 2025 indicates that the thematic progression of SFRT followed a distinct three-phase temporal pattern (34). Keyword cluster mapping (**Figure 7A**) and the identification of top burst keywords (**Figure 7B**) delineate these specific pathways. This macroscopic thematic evolution across the three temporal phases is further visualized in **Figure 8**. During the initial phase (2005–2015), the literature prominently cataloged foundational physics and preclinical models (25). Keywords including “microbeam,” “synchrotron,” “x-rays,” “dosimetry,” and specific terms like “9L gliosarcoma” and “brain” (Red Cluster) exhibited the highest occurrence frequencies across the dataset.

3.7.2 Between 2016 and 2022, keyword distributions demonstrated a broad volume increase and technological diversification. Terms originating from the Green and Yellow Clusters, such as “grid therapy,” “SFRT,” “lattice radiotherapy,” “immunotherapy,” “bystander,” “lung-cancer,” and “Phase I trial,” achieved prominence (24, 35, 36). Simultaneously, keywords including “Proton Minibeam Radiation Therapy” and “Monte Carlo simulations” from the Light Blue Cluster progressed in specific sub-domains (37), aligning with an expansion into complex biological modalities in the indexed literature (16, 33).

In the current phase (2023–2025), burst keywords such as “management,” “optimization,” and “Phase I trial” sustained high occurrence frequencies within the Green and Yellow Clusters, alongside the emergence of “TOPAS” (a mapped dose calculation software) within the Light Blue Cluster (31, 38, 39). Concurrently, the Purple Cluster, characterized by the foundational keywords “cells” and “ionizing-radiation,” maintained a consistent presence (18, 32), reflecting continuous baseline data generation corresponding to these temporal phases (16).

## 4 Discussion

### 4.1 General information and citation landscape

This comprehensive bibliometric analysis, encompassing 427 documents published from 2005 to 2025, unequivocally establishes Spatially Fractionated Radiotherapy (SFRT) as a consistently evolving and inherently interdisciplinary domain. The publication trajectory exhibits a clear three-phase evolution: an initial dormant period (2005–2014), a steady development phase (2015–2019), and a substantial acceleration from 2020 onward, indicating a shift toward clinically focused innovations (4, 40).

The geographic composition reveals that the United States contributes the highest research volume, while France demonstrates exceptional citation impact, reflecting diverse national research strategies (41). The broad dissemination of SFRT research across 92 journals emphasizes the complex technical and biological integration required between medical physics and oncology (4, 42). While such cross-disciplinary synergy catalyzes innovation, it simultaneously poses challenges for clinical standardization (41, 43).

The co-citation landscape further illustrates how foundational works shape current translational oncology. For instance, the seminal introduction of GRID technology (44), the fundamental preclinical validation in animal models (34), and subsequent comprehensive spatial fractionation evaluations (1) form the critical conceptual basis bridging the historical origins of the technique with modern therapeutic applications.

### 4.2 Advances in SFRT technologies and therapeutic strategies

The knowledge structure of SFRT research relies on distinct physical and biological pillars. Cluster 1 establishes the technological basis of Microbeam Radiation Therapy (MRT), focusing on dosimetry and initial preclinical evaluations in models such as 9L gliosarcoma (19, 45). This ensures accurate radiation delivery (46, 47) and establishes early biological frameworks (18). Equivalently, Cluster 5 addresses the core cellular-level biological interactions with ionizing radiation, underpinning all SFRT modalities (32). This fundamental science remains the ultimate pillar for understanding how to maximize targeted cell death while minimizing normal tissue damage (6, 17), driving the continuous resolution of complex clinical challenges (16, 48).

Building upon these foundational tools, Cluster 2 encompasses broader spatial fractionation modalities, notably GRID and LATTICE radiotherapy (21). Moving beyond purely physical ablation, this domain primarily investigates the biological modulation of host responses, heavily emphasizing immunotherapy and the bystander effect (3, 24, 49). The advancement of these modalities into Phase I trials, particularly for malignancies like lung cancer, signifies a vital translational leap (50–52), confirming the capacity of dose heterogeneity to modulate the tumor microenvironment in human applications (36, 53).

Clusters 3 and 4 represent the latest push toward computational precision and clinical optimization. Cluster 3 signifies the advanced development of Proton Minibeam Radiation Therapy (pMBRT) (27), utilizing analytical calculations and Monte Carlo simulations for supreme dosimetric precision (26, 28, 29, 54), often validated within specialized *in vivo* models (25). This leverages state-of-the-art physics for highly controllable treatments (37, 55, 56). Consequently, Cluster 4 focuses on evaluating the absolute clinical feasibility and methodological optimization of these precise fractionations (31, 38), addressing the practical considerations of translation (27, 30).

### 4.3 Technical constraints and limitations in dose optimization

Despite immense theoretical promise, the systemic optimization of SFRT is presently constrained by intertwined technical and biological impediments (48, 57). Resolving these hurdles requires intense multidisciplinary convergence (42, 43).

A primary limitation is the difficulty of integrating dynamic biological factors into physical dose planning. Current methodologies cannot easily incorporate complex intra-tumoral characteristics—such as hypoxia, rapid proliferation, or immune microenvironment variations—to optimally place “peak” doses (36, 58). Furthermore, radiation oncology lacks robust predictive radiobiological models capable of forecasting non-targeted immune activation or normal tissue tolerance under extreme gradients (49, 50). Therefore, determining precise peak-to-valley dose ratios or spatial distributions remains largely empirical rather than objectively calculated (34, 59).

Concurrently, high-precision delivery demands highly advanced collimator systems to shape intricate gradients (8, 60, 61). During treatment, physiological organ motion (e.g., respiration) severely threatens the fidelity of these extreme distributions, necessitating superior real-time adaptive systems (62). Traditional quality assurance systems are frequently inadequate for verifying sub-millimeter heterogeneous plans (63, 64), requiring novel dosimetric methods (46, 47) and unified international guidelines for distinct modalities (21, 41, 44).

Biologically, the exact trigger thresholds for bystander and abscopal mechanisms remain partially quantified (3), demanding sophisticated *in vivo* models for accurate assessment (58, 65). The lack of reliable clinical biomarkers complicates patient stratification and response prediction (16, 24). Finally, the relatively limited volume of large-scale, prospective clinical trials limits long-term efficacy validation (48, 66, 67), severely delaying regulatory standardization and widespread adoption (40, 41, 43).

### 4.4 Systemic evolution: integrating dose heterogeneity and radiobiology

Validated by the three-phase thematic map evolution, the historical trajectory of SFRT reflects a progressive solution to the core oncological dilemma: maximizing malignant tissue ablation while meticulously sparing functional organ structures (2, 5).

The initial research phase (2005–2015) successfully established the mechanics of spatial macro-fractionation (19, 45). This period prioritized deliberate dose redistribution (2) through verified micro-dosimetry (46, 47), successfully establishing partial-volume normal tissue tolerance in sensitive targets such as the brain (32).

The intermediate development phase (2016–2022) marked the integration of these mechanics with complex biological responses (16, 26, 27). Research extensively targeted non-targeted antitumor mechanisms (58), particularly investigating how “valley” sub-volumes mediate survival signals or distress indicators to adjacent “peak” regions (58). This era underscored the utilization of immunogenic cell death to provoke systemic abscopal phenomena (68, 69).

The recent expansion phase (2023–2025) shifts toward standardization and technical precision (31, 38). Through superior delivery algorithms and simulation platforms (e.g., Monte Carlo, TOPAS) (28, 29), the current focus is refining the overarching therapeutic index (5, 17). Translating high efficacy without generating severe toxicities is the premier objective (52, 67, 70). Emphasizing continuous investigation into fundamental cellular ionizing interactions (6, 32, 71) ensures that the clinical utilization of dose arrays remains firmly anchored in established radiobiology, solidifying SFRT’s status as a premier therapy for heterogeneous masses (48).

### 4.5 Limitations and future directions

The bibliometric method incorporates certain inherent limitations. Relying strictly on specific indices (even multiple like WoSCC and PubMed) may still inadvertently bypass adjacent biomedical literature (47). Nomenclature inconsistencies in an evolving field also create retrieval liabilities (48). Most critically, there is a pronounced translational gap, as much literature resides in the Phase I or pre-clinical sphere, minimizing definitive outcome data (67) and limiting direct guidelines for end-stage patients (16). Geographic imbalance likewise stymies global standard consensus (41, 43), and standardizing technical comparisons across significantly differing physics platforms remains challenging (4, 21).

To maximize SFRT’s utility beyond end-stage palliation (48), future investigations must clarify underlying mechanisms (57). Specifically, pharmacologically or genetically regulating bystander (3, 65) and abscopal immune networks via combinations with targeted checkpoint therapies should be prioritized (33). Enhancing precise dose planning algorithmically via functional imaging (72), utilizing dynamic motion-tracking and heavy ion techniques (65, 66), are essential. Discovering reliable prognostic biomarkers and predictive models (16, 24, 73) and analyzing systemic pharmacological synergies (74) within the framework of rigorous, randomized, multi-center trials (59, 69) represent the ultimate path forward.

### 4.6 Clinical takeaway

The transition of SFRT from physics-based mathematical models to actionable clinical pathways confirms its utility in managing radioresistant, bulky malignancies (typically >8 cm) where homogenous maximum tolerated doses structurally fail. Utilizing evidence-based modalities like GRID and LATTICE, clinicians now possess tools mapping functional palliation and, occasionally, synergistic curative potential when combined with immunotherapy (75, 76).

4.6.2 For direct clinical application, patient stratification is imperative. Care providers should specifically screen for tumors exhibiting rapid proliferation or centralized hypoxia, as extreme intra-tumoral dose heterogeneity fundamentally leverages these aggressive characteristics to induce vascular collapse and antigenic shedding. Integrating PET/CT functional tracking to position “ablative peaks” strictly over highly metabolically active sub-volumes ensures that spatial manipulation translates into definitive tumor regression and symptom resolution without sacrificing adjunctive critical organs.

### 4.7 Comparative advantages of this study

A recent literature search utilizing the core terms “Radiotherapy” and “bibliometric” in relevant databases yields over 260 existing studies, indicating the robust application of this methodology within radiation oncology. A synthesis of this vast corpus reveals that previous bibliometric works have predominantly focused on macroscopic technological shifts (such as SBRT, FLASH radiotherapy, and heavy-ion therapy), specific oncological paradigms (e.g., glioma, prostate cancer), or broad interdisciplinary integrations like artificial intelligence and radiomics. Additionally, extensive literature has mapped the management of radiation-induced toxicities and academic impact metrics.

While these existing studies provide valuable macroscopic and disease-specific overviews, there is a pronounced lack of specialized, granular bibliometric mapping dedicated exclusively to SFRT. This study addresses this critical literature gap.

Compared to existing broad-scope radiotherapy bibliometrics, our research exhibits several distinct advantages. First, it offers a highly focused, two-decade longitudinal mapping that uniquely captures the paradigm shift from purely physical “anatomical ablation” (traditional GRID) towards “biological targeting and systemic immunomodulation” uniquely achieved by SFRT. Second, by structurally analyzing the technological convergence from macroscopic LATTICE to advanced microscopic Proton Minibeam Radiation Therapy (pMBRT), this study pinpoints a highly specialized technological frontier that generalized radiotherapy bibliometrics intrinsically overlook. Finally, the synergistic application of three distinct analytical platforms, complete with standardized keyword consolidation and parameter transparency, provides a highly reproducible structural validation of thematic evolution, establishing an optimal reference model for future sub-disciplinary bibliometric investigations.

## 5 Conclusions

This bibliometric analysis, spanning 2005–2025, provides the first comprehensive overview of SFRT. It reveals SFRT as a progressively growing, interdisciplinary field experiencing substantial publication and citation volume increases, particularly since 2020. This trajectory highlights SFRT’s emergence as a paradigm shift in radiation oncology, leveraging controlled intra-tumoral dose heterogeneity and radiobiological interactions. The research evolved through three distinct phases, driven by five core clusters: Cluster 1 (Microbeam Technology) and Cluster 5 (Fundamental Radiobiology) established early physical principles; Cluster 2 (Broad SFRT Modalities, Immune Modulation, Clinical Translation) advanced radiobiological mechanisms and early clinical applications; while Cluster 3 (Advanced Proton SFRT, Computational Precision) and Cluster 4 (Methodological Optimization, Clinical Feasibility) spearheaded recent precision and optimization efforts. This integrated evolution of physical and biological principles is key to SFRT’s therapeutic efficacy.

To fully realize SFRT’s potential, future endeavors must prioritize deepening biological understanding, developing advanced delivery technologies, identifying robust biomarkers, and expanding clinical applications through rigorous trials. By focusing on these strategic directions, SFRT is poised to become a cornerstone in treating challenging malignancies, ultimately improving patient outcomes.

## Data Availability

The original contributions presented in the study are included in the article/**Supplementary Material**. Further inquiries can be directed to the corresponding author.

## Supplementary material

Supplementary Table 1 The rationale and details of keyword consolidation and standardization prior to bibliometric co-occurrence analysis.

Supplementary Table 2 Detailed configuration parameters used in the multi-platform bibliometric analysis to ensure methodological transparency and reproducibility.

Supplementary Table 3 The top 10 co-authors related to SFRT.

Supplementary Table 4 The top 10 co-cited references related to SFRT.

Supplementary Table 5 The top 50 keywords related to SFRT.
