# Supplementary material for: Bibliometric analysis of global research trends in spatially fractionated radiotherapy
Source: Front Oncol. 2026 May 8;16:1828039. doi: 10.3389/fonc.2026.1828039 (PMC13193894; doi:10.3389/fonc.2026.1828039)
Supplement: Supplementary Table 1 — The rationale and details of keyword consolidation and standardization prior to bibliometric co-occurrence analysis. [file Table1.docx]

**Supplementary Table 1:** The rationale and details of keyword consolidation and standardization prior to bibliometric co-occurrence analysis.

| **Standardized Keyword (Target Term)** | **Original Raw Keywords Synthesized (Source Terms)** |
| --- | --- |
| Spatially fractionated radiotherapy | spatially fractionated radiation therapy; sfrt; spatial fractionation; spatially fractionated radiotherapy; spatially-fractionated radiation |
| GRID therapy | grid radiation therapy; grid radiotherapy; grid; spatial grid radiotherapy; 2d grid therapy |
| LATTICE radiotherapy | lattice radiation therapy; lrt; lattice spatially fractionated radiation therapy; lattice therapy |
| Microbeam radiation therapy | microbeam radiotherapy; mrt; microbeam radiation; synchrotron microbeam radiation therapy |
| Proton minibeam radiation therapy | proton minibeam radiotherapy; pmbrt; minibeam radiation therapy; proton minibeams |
| Bystander effect | bystander effects; radiation-induced bystander effect; radiation induced bystander effects; bystander signaling |
| Stereotactic body radiotherapy | sbrt; stereotactic body radiation therapy; stereotactic ablative radiotherapy; sabr |
| Tumor microenvironment | tumour microenvironment; tumor micro-environment; tme; microenvironment |
| Squamous cell carcinoma | squamous cell carcinomas; scc; squamous-cell carcinoma |
| Monte Carlo simulation | monte carlo; monte-carlo simulations; monte carlo calculations |

**Supplementary Table 2:** Detailed configuration parameters used in the multi-platform bibliometric analysis to ensure methodological transparency and reproducibility.

| **Analytical Platform** | **Analytical Module / Output** | **Key Configuration Parameters & Thresholds** |
| --- | --- | --- |
| VOSviewer (v1.6.17) | Country Co-authorship | Counting method: Full counting; Minimum number of documents per country = 4; Network layout: Attraction = 1, Repulsion = -3. |
|  | Institution Co-authorship | Counting method: Full counting; Minimum number of documents per institution = 5; Network layout: Attraction = 1, Repulsion = -2. |
|  | Journal/Co-cited Journal | Minimum number of documents per journal = 5; Minimum co-citations per journal = 100; Network layout: Attraction = 2, Repulsion = -2. |
|  | Author/Co-cited Author | Minimum documents per author = 8; Minimum co-citations per author = 50; Network layout: Attraction = 1, Repulsion = -6 (Author) / Attraction = 2, Repulsion = 0 (Co-cited). |
|  | Reference Co-citation | Minimum number of co-citations per reference = 50; Network layout: Attraction = 1, Repulsion = -1. |
|  | Keyword Co-occurrence | Minimum number of occurrences per keyword = 15; Network layout: Attraction = 1, Repulsion = -1. |
| CiteSpace (v6.1.R6) | Dual-map Overlay (Journals) | Time Slicing: 2005 to 2025 (Years per slice = 1); Node Types: Journal; Base map: default Blondel algorithm; Z-score visualization applied. |
|  | Citation Burst (References) | Time Slicing: 2005 to 2025; Node Types: Cited Reference; Selection Criteria: g-index (k = 25); Pruning: Pathfinder, Pruning sliced networks. |
|  | Citation Burst (Keywords) | Time Slicing: 2015 to 2025 (adjusted for keyword focus); Node Types: Keyword; Selection Criteria: g-index (k = 5); Pruning: Pathfinder, Pruning sliced networks. |
| Biblioshiny (v5.2.0) | Thematic Map & Evolution | Time-span: 2005 to 2025; Field: Keywords Plus / Author Keywords; Number of words: Top 250; Minimum cluster frequency: 5; Weighting: Inclusiveness (Walktrap algorithm). |
|  | Geographic Mapping | Spatial projection: OpenStreetMap integration; Threshold: minimum 1 publication for coloring. |

**Supplementary Table 3:** The top 10 co-authors related to SFRT.

| **Rank** | **Co-cited Author** | **Citations** |
| --- | --- | --- |
| 1 | Prezado, Y. | 667 |
| 2 | Dilmanian, F. A. | 366 |
| 3 | Mohiuddin, M. | 301 |
| 4 | Bouchet, A. | 213 |
| 5 | Laissue, J. A. | 183 |
| 6 | Amendola, B. E. | 177 |
| 7 | Zhang, H. L. | 153 |
| 8 | Serduc, R. | 145 |
| 9 | Slatkin, D. N. | 127 |
| 10 | Fernandez-Palomo, C. | 126 |

**Supplementary Table 4:** The top 10 co-cited references related to SFRT.

| **Rank** | **Co-cited reference** | **Author** | **Year** | **DOI** | **Citations** | **Title** |
| --- | --- | --- | --- | --- | --- | --- |
| 1 | International Journal of Radiation Oncology Biology Physics | Mohiuddin, M. | 1999 | 10.1016/S0360-3016(99)00170-4 | 140 | High-dose spatially-fractionated radiation (GRID): a new paradigm in the management of advanced cancers |
| 2 | Neuro-Oncology | Dilmanian, F. A. | 2002 | 10.1093/NEUONC/4.1.26 | 94 | Response of rat intracranial 9L gliosarcoma to microbeam radiation therapy |
| 3 | International Journal of Radiation Oncology Biology Physics | Billena, C. | 2019 | 10.1016/J.IJROBP.2019.01.073 | 88 | A Current Review of Spatial Fractionation: Back to the Future? |
| 4 | Clinical and Translational Radiation Oncology | Yan, W. | 2020 | 10.1016/J.CTRO.2019.10.004 | 88 | Spatially fractionated radiation therapy: History, present and the future |
| 5 | Proceedings of the National Academy of Sciences of the United States of America | Dilmanian, F. A. | 2006 | 10.1073/PNAS.0603567103 | 85 | Interlaced x-ray microplanar beams: a radiosurgery approach with clinical potential |
| 6 | Medical Physics | Prezado, Y. | 2013 | 10.1118/1.4791648 | 82 | Proton-minibeam radiation therapy: a proof of concept |
| 7 | Cancer | Mohiuddin, M. | 1990 | 10.1002/1097-0142(19900701)66 | 78 | Palliative treatment of advanced cancer using multiple nonconfluent pencil beam radiation. A pilot study |
| 8 | International Journal of Radiation Oncology Biology Physics | Neuner, G. | 2012 | 10.1016/J.IJROBP.2011.01.065 | 77 | High-dose spatially fractionated GRID radiation therapy (SFGRT): a comparison of treatment outcomes with Cerrobend vs. MLC SFGRT |
| 9 | International Journal of Radiation Oncology Biology Physics | Peñagarícano, J. A. | 2010 | 10.1016/J.IJROBP.2009.03.030 | 76 | Evaluation of spatially fractionated radiotherapy (GRID) and definitive chemoradiotherapy with curative intent for locally advanced squamous cell carcinoma of the head and neck: initial response rates and toxicity |
| 10 | Scientific Reports | Prezado, Y. | 2017 | 10.1038/S41598-017-14786-Y | 76 | Proton minibeam radiation therapy spares normal rat brain: Long-Term Clinical, Radiological and Histopathological Analysis |

**Supplementary Table 5:** The top 50 keywords related to SFRT.

| **Rank** | **Keywords** | **Counts** | **Rank** | **Keywords** | **Counts** |
| --- | --- | --- | --- | --- | --- |
| **1** | radiotherapy | 129 | **26** | 9l gliosarcoma | 21 |
| **2** | radiation-therapy | 93 | **27** | feasibility | 21 |
| **3** | irradiation | 74 | **28** | optimization | 21 |
| **4** | dosimetry | 56 | **29** | ray microplanar beams | 21 |
| **5** | cancer | 44 | **30** | microbeam radiotherapy | 20 |
| **6** | grid therapy | 43 | **31** | x-rays | 20 |
| **7** | spatially fractionated radiation therapy | 40 | **32** | glioma-bearing rats | 19 |
| **8** | therapy | 38 | **33** | grid | 19 |
| **9** | cells | 35 | **34** | model | 19 |
| **10** | spatially fractionated radiotherapy | 35 | **35** | beams | 18 |
| **11** | lung-cancer | 32 | **36** | ionizing-radiation | 18 |
| **12** | bystander | 30 | **37** | lattice radiotherapy | 18 |
| **13** | microplanar beams | 29 | **38** | minibeam radiation therapy | 18 |
| **14** | sfrt | 29 | **39** | topas | 18 |
| **15** | microbeam | 28 | **40** | microbeam radiation therapy | 17 |
| **16** | synchrotron | 26 | **41** | monte carlo | 17 |
| **17** | beam | 24 | **42** | spatial fractionation | 17 |
| **18** | brain | 24 | **43** | efficacy | 16 |
| **19** | immunotherapy | 24 | **44** | proton minibeam radiation therapy | 16 |
| **20** | radiation | 24 | **45** | tumor | 16 |
| **21** | microbeam radiation-therapy | 23 | **46** | phase-i trial | 15 |
| **22** | management | 22 | **47** | mrt | 14 |
| **23** | monte carlo simulations | 22 | **48** | sfgrt | 14 |
| **24** | proton therapy | 22 | **49** | survival | 14 |
| **25** | tolerance | 22 | **50** | advantage | 13 |
