# Supplementary figures and images for: Bibliometric analysis of global research trends in spatially fractionated radiotherapy
Source: Front Oncol. 2026 May 8;16:1828039. doi: 10.3389/fonc.2026.1828039 (PMC13193894; doi:10.3389/fonc.2026.1828039)

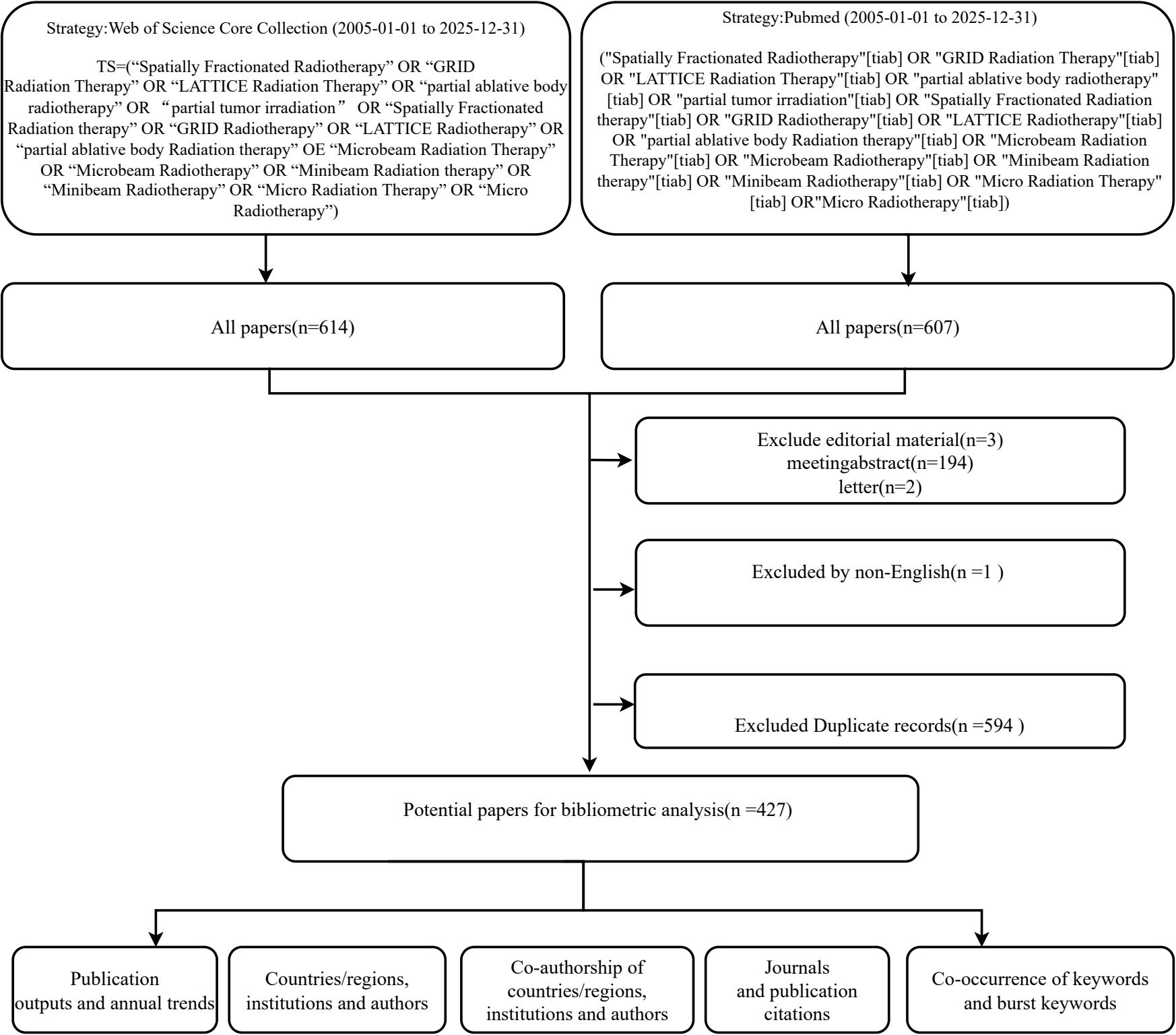

Supplement: Supplementary file 2 [file Image1.pdf]
